# Myopic Shift and Outdoor Activity among Primary School Children: One-Year Follow-Up Study in Beijing

**DOI:** 10.1371/journal.pone.0075260

**Published:** 2013-09-24

**Authors:** Yin Guo, Li Juan Liu, Liang Xu, Ping Tang, Yan Yun Lv, Yi Feng, Meng Meng, Jost B. Jonas

**Affiliations:** 1 Tongren Eye Care Center, Beijing Tongren Hospital, Capital Medical University, Beijing, China; 2 Beijing Institute of Ophthalmology, Beijing Tongren Hospital, Capital Medical University, Beijing, China; 3 Department of Ophthalmology, Medical Faculty Mannheim of the Ruprecht-Karls-University Heidelberg, Germany; Institut de la Vision, France

## Abstract

**Purpose:**

To assess whether a change in myopia related oculometric parameters of primary school children in Beijing was associated with indoors and outdoors activity.

**Methods:**

The longitudinal school-based study included school children who were examined in 2011 and who were re-examined in 2012. The children underwent a comprehensive eye examination including ocular biometry by optical low-coherence reflectometry and non-cycloplegic refractometry. Parents and children had a detailed interview including questions on time spent indoors and outdoors.

**Results:**

Out of 681 students examined at baseline, 643 (94.4%) returned for follow-up examination. Within the one-year period, mean time spent daily outdoors increased by 0.4±0.9 hours, mean axial length by 0.26±0.49 mm, the ratio of axial length divided by anterior corneal curvature (AL/CC) by 0.03±0.06, and myopic refractive error by −0.06±0.89 diopters. In multivariate analysis, elongation of axial length was significantly associated with less total time spent outdoors (P = 0.02; standardized coefficient beta −0.12) and more time spent indoors with studying (P = 0.007; beta: 0.14) after adjustment for maternal myopia (P = 0.02; beta: 0.12). An increase in AL/CC was significantly associated with less time spent outdoors (P = 0.01; beta:−0.12) after adjustment for paternal myopia (P = 0.003; beta: 0.15) and if region of habitation was excludedors for leisure (P = 0.006; beta:−0.13), with less total time spent outdoors (P = 0.04; beta:−0.10), or with more time spent i. An increase in myopic refractive error, after adjustment for age, was significantly associated with less time spent outdo ndoors with studying (P = 0.005; beta: 0.13).

**Conclusions:**

A change in oculometric parameters indicating an increase in myopia was significantly associated with less time spent outdoors and more time spent indoors in school children in Greater Beijing within a study period of one year. Our study provides additional information on the potentially helpful role of outdoors activity in the prevention of myopia. Public health care measures such as school agendas may potentially take it into account.

## Introduction

Myopia related changes in the macula and optic nerve head belong to the most common causes for visual impairment and visual field defects in elderly Chinese [1], [2]. Recent studies have evidenced a major myopic shift in the young generation in East and Southeast Asian countries including China [3], [4]. These studies suggested a future increase in myopia related visual impairment in these countries. Knowledge about modifiable factors in the development of myopia is therefore of high importance for public health, in particular in East Asian and Southeast Asian countries. Numerous studies have previously examined a reverse relationship between the amount of outdoor activities or the time spent outdoors and the development of myopia in children [5]–[11]. Besides these cross-sectional investigations, other studies examined longitudinally the association between parameters of outdoors activities and myopia [12]–[23]. These longitudinal studies were carried in different countries with children of varying ethnic background. Only few of these longitudinal studies were performed on Chinese children, with one study from Taiwan and one study from South China [19], [22]. We therefore performed a longitudinal study on students in Beijing to address the question whether a change in myopia is related with outdoor/indoors activities after adjusting for general parameters such as the socioeconomic background of the parents.

## Methods

### Ethics Statement

The study protocol was approved by the Human Research Ethics Committee of the TongRen Hospital, Capital Medical University Beijing. After explanation of the study design to parents and children, informed written consent was obtained from at least one parent.

The school-based study was initially carried out in 2011, and the follow-up study was performed one year later in 2012 [24]. At baseline, 681 subjects from grade-1 and grade-4 were included. The study was divided into a rural part (311 individuals; 138 girls) and an urban part (370 individuals; 166 girls). The mean age of the children was 7.7±1.6 years (median: 7 years; range: 5–13 years).

At baseline and at the follow-up examination, a comprehensive eye examination was carried out, including measurement of visual acuity, auto-refractometry, assessment of ocular motility, slit-lamp examination of the anterior ocular segment, and non-mydriatic digital fundus photography (45°; CR-DGI camera, Canon Inc, Tokyo, Japan). Visual acuity was measured by an ophthalmic technologist who used a retro-illuminated ETDRS (Early Treatment of >Diabetic Retinopathy Study) chart with tumbling-E optotypes. Ocular biometric parameters (central corneal thickness, corneal curvature, anterior chamber depth, lens thickness, axial length) were measured for the right eye of all subjects by optical low-coherence reflectometry (Lensstar 900® Optical Biometer, Haag-Streit, 3098 Koeniz, Switzerland). We additionally calculated the ratio of axial length divided by anterior corneal curvature (AL/CC). The AL/CC ratio, also known as the AL/CR (axial length/corneal radius) ratio, has been used as surrogate for refractive error. Since it includes corneal refractive power, it is a better correlate for refractive error than it is axial length as single parameter [25]. Refractometry was performed in a non-cycloplegic state by auto-refractometry (auto-refractor KR-8900, Topcon, Tokyo, Japan) followed by subjective refractometry. The results of the subjective refractometry based on the automatic refractometry were taken for further analysis. The examination was performed so long until a reliable result was obtained. The spherical equivalent of the refractive error was calculated as spherical refractive error +1/2 cylindrical refractive error. All examinations were undertaken by trained ophthalmologists and optometrists. Refractometry was not performed in cycloplegia since a relatively large number of parents would not have accepted cycloplegia, so that the study sample might no longer have been representative. Since we carried out biometry and calculated the AL/CC ratio as surrogate for refractive error, we considered the advantage of having a relatively high participation rate being higher than the disadvantage of an examination without cycloplegia.

The parents underwent an interview by trained and supervised interviewers with questions on how long the children needed to go to school and to return home; what kind of transportation (walking, bicycle, private car, public transport) was used; how long the children spent outdoors during school breaks; what kind of sport and how long they performed it outdoors during the week and the weekends; and what other activities the children had outdoors and how long they had it, such as playing outdoors, having picnics, or walking. The interview additionally included questions about the time used to study indoors, about the time of watching television, and the time to play with electronic devices indoors. The same questionnaire had been used in a previous interview held at the baseline examination of the study [24]. The average number of daily outdoor activity hours and indoor activity hours was calculated using the formula: [(hours spent on a weekday)×5+ (hours spent on a weekend day) ×2]/7. The total outdoor activity was defined as the sum of outdoor leisure and outdoor sports.

The statistical analysis was performed using a commercially available statistical software package (SPSS for Windows, version 20.0, SPSS, Chicago, IL). To examine the possible impact of nonparticipation at the follow-up examination, sociodemographic and clinical characteristics were compared between participants who completed the follow-up examination and non-participants of the follow-up examination. The parameters were presented as mean ± standard deviation. To examine the associations between elongation of axial length and other parameters, the *chi-square* test was applied for categorical variables and a linear regression analysis for continuous variables. After univariate analysis of potential associations, we performed a stepwise multivariate analysis with the change in myopia related oculometric parameters (i.e. axial length, AL/CC, refractive error) as dependent variables, and all parameters as independent variables which showed a significant association with the main parameters in univariate analysis. In a stepwise manner, we then dropped all parameters from the list of independent variables when they were no longer significantly associated with the dependent parameter. The baseline parameters of activity measures were used for the statistical analysis. Odds ratios (OR) were calculated and 95% confidence intervals (CI) were presented. All *P*-values were 2-sided and were considered statistically significant when the values were less than 0.05. Only measurements of the right eyes were taken for the statistical analysis.

## Results

In 2012, 643 (94.4%) out of the 681 students examined at baseline returned for the follow-up examination, whereas 6 (0.9%) students were not at school during the examination day and 32 (4.7%) students had entered other schools during the follow-up period. In 2011, the mean age of these 681 participants was 7.7±1.6 years (range: 5–13 years) with 54.3% of them living in the urban area; 56.1% of them were from grade-1, and 44.6% of them were girls. Non-participants as compared with the participants were predominantly boys and lived predominantly in the rural region (*P* = 0.001); both groups did not differ significantly in age (7.7±1.6 years versus 8.1±2.0 years; *P* = 0.13).

**Figure 1:**
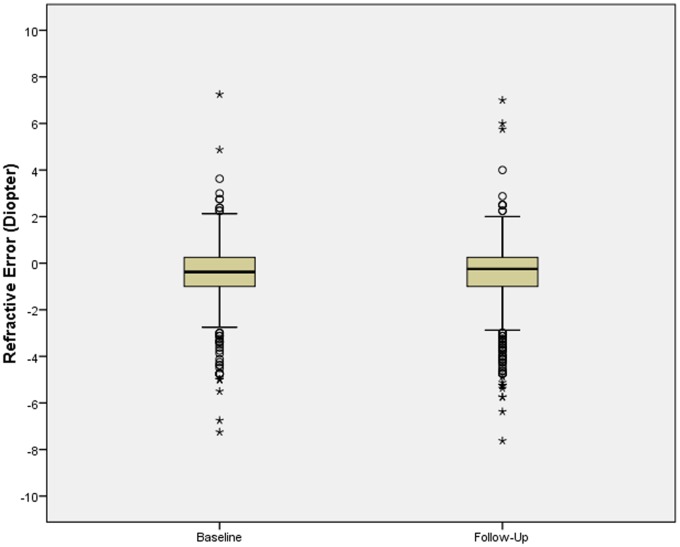
Boxplot Showing the Distribution of Refractive Error at Baseline and Follow-Up of the Study.

**Figure 2:**
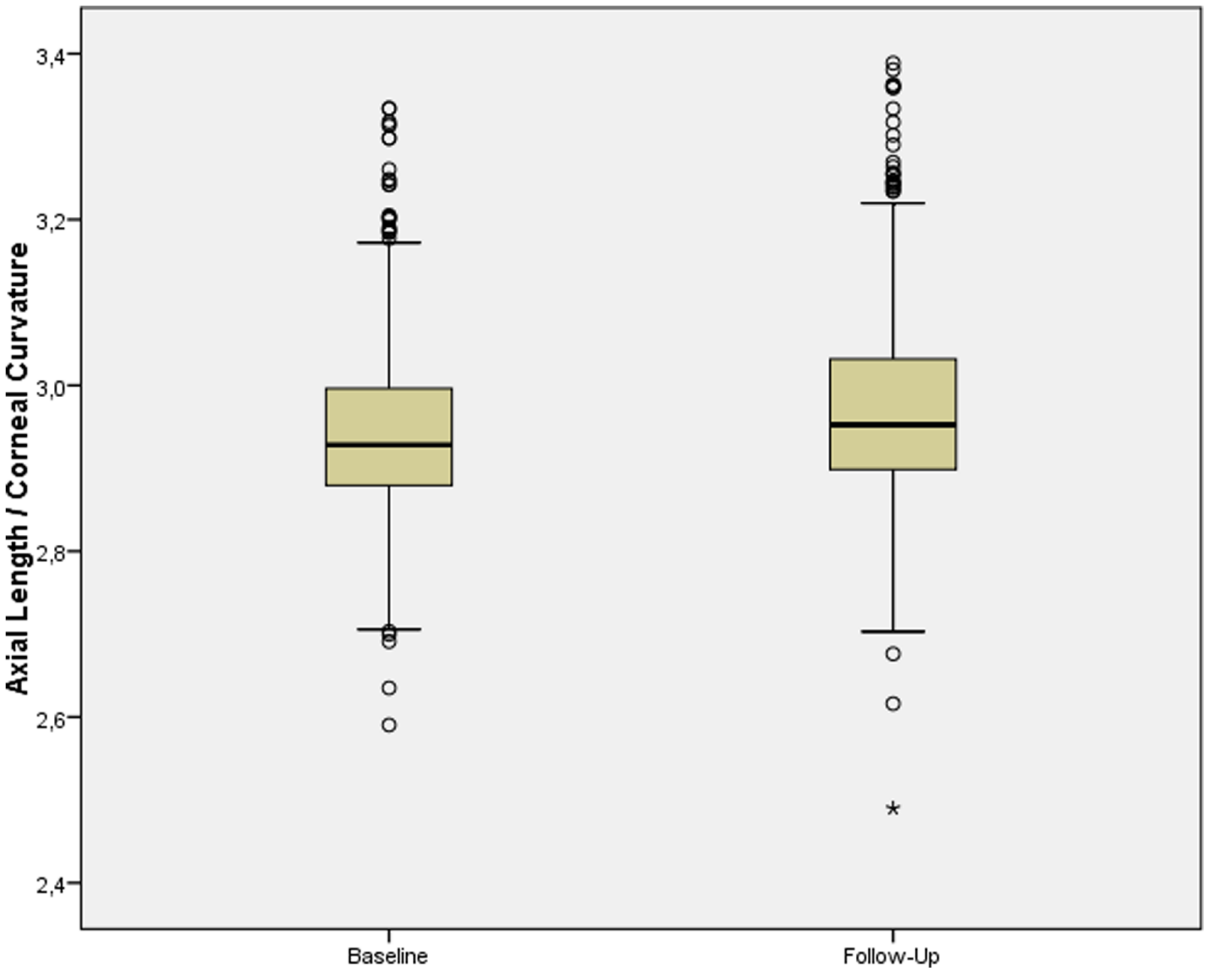
Boxplot Showing the Distribution of the Ratio of Axial Length to Corneal Curvature at Baseline and Follow-Up of the Study.

In the 2012 survey, the mean refractive error (−0.62±1.49 diopters) had changed by −0.06±0.89 diopters, mean axial length (23.29±1.02 mm) had elongated by 0.26±0.49 mm, and the mean AL/CC ratio (2.98±0.12) had increased by 0.03±0.06 (Table 1) (Fig. 1, 2). The mean time spent daily outdoors (2.0±0.8 hours) had increased by 0.4±0.9 hours, the mean time spent daily indoors with studying (5.5±0.9 hours) had increased by 0.2±1.0 hours, and the mean time spent daily with playing electronic gadgets (0.4±0.5 hours) had increased by 0.3±0.5 hours. The mean time spent daily on watching television (0.9±0.9 hours) had remained unchanged (Table 1).

### Elongation in Axial Length

**Table 1:** Characteristics of the Study population at Baseline in 2011 and Follow-Up in 2012, Stratified by School Grade.

	All Study Participants		Grade 2 Students		Grade 5 Students	
Year	2011	2012	2011	2012	2011	2012
Axial Length (mm)	23.03±0.96 (median: 22.99; 95%CI*: 21.31, 25.15)	23.29±1.02 (median: 23.24; 95%CI*: 21.46, 25.54)	22.69±0.79 (median: 23.70; 95%CI: 21.20, 24.36)	22.97±0.83 (median: 23.00; 95%CI: 21.39, 24.68)	23.46±1.00 (median: 23.37; 95%CI: 21.59, 25.60)	23.69±1.10 (median: 23.54; 95%CI: 21.61, 26.22)
Axial Length/Corneal Curvature	2.94±0.11 (median: 2.93; 95%CI: 2.75, 3.20)	2.98±0.12 (median: 2.95; 95%CI: 2.78, 3.25)	2.90±0.08 (median: 2.90; 95%CI: 2.73, 3.08)	2.93±0.09 (median: 2.93; 95%CI: 2.77, 3.15)	3.00±0.11 (median: 2.99; 95%CI: 2.79, 3.29)	3.03±0.13 (median: 3.01; 95%CI: 2.81, 3.34)
Refractive Error (Diopters) Right Eye	−0.55±1.31 (median: −0.38; 95%CI: −4.18, +1.50)	−0.62±1.49 (median: −0.25; 95%CI: −4.62, +1.53)	−0.22±1.00(median: −0.19; 95%CI: −2.50, +1.86)	−0.16±0.98 (median: −0.06; 95%CI: −2.25, +1.77)	−0.96±1.51 (median: −0.75; 95%CI: −4.75, +1.00)	−1.22±1.80 (median: −0.75; 95%CI: −5.25, +0.90)
Outdoor Activities (hours daily)	1.62±0.80 (median: 1.41; 95%CI: 0.59, 3.78)	2.05±0.78 (median: 1.86; 95%CI: 1.14, 3.97)	1.63±0.75 (median: 1.48; 95%CI: 0.73, 3.85)	1.98±0.64 (median: 1.86; 95%CI: 1.14, 3.68)	1.60±0.85 (median: 1.37; 95%CI: 0.49, 3.67)	2.14±0.91 (median: 1.89; 95%CI: 1.14, 4.64)
Outdoor Sports (hours daily)	0.67±0.14 (median: 0.66; 95%CI 0.49, 1.01)	1.02±0.43 (median: 0.95; 95%CI 0.28, 2.08)	0.69±0.11 (median: 0.69; 95%CI: 0.59, 1.01)	0.98±0.38 (median: 0.95; 95%CI: 0.37, 1.84)	0.65±0.16 (median: 0.66; 95%CI: 0.49, 1.05)	1.06±0.48 (median: 0.99; 95%CI: 0.28, 2.28)
Outdoor Leisure (hour daily)	0.98±0.81 (median: 0.71; 95%CI: 0.00, 3.09)	1.03±0.60 (median: 0.86; 95%CI: 0.36, 2.58)	1.01±0.81 (median: 0.83; 95%CI: 0.03, 3.19)	1.00±0.51 (median: 0.84; 95%CI: 0.42, 2.46)	0.94±0.81 (median: 0.60; 95%CI: 0.00, 2.95)	1.08±0.69(median: 0.93; 95%CI: 0.36, 3.27)
Studying Time (hours daily)	5.26±0.89 (median: 5.31; 95%CI: 3.37, 6.88)	5.50±0.92 (median: 5.52; 95%CI: 3.69, 7.31)	5.14±0.85 (median: 5.23; 95%CI: 3.16, 6.78)	5.45±0.79 (median: 5.49; 95%CI: 3.81, 7.13)	5.39±0.92 (median: 5.52; 95%CI: 3.46, 7.08)	5.57±1.08 (median: 5.62; 95%CI: 3.69, 7.62)
Watching Television (hours daily)	0.90±0.97 (median: 0.57; 95%CI: 0.00, 0.99)	0.89±0.91 (median: 0.57; 95%CI: 0.00, 3.57)	0.81±0.85 (median: 0.57; 95%CI: 0.00, 3.10)	0.90±0.81 (median: 0.57; 95%CI: 0.00, 2.93)	1.01±1.10 (median: 0.57; 95%CI: 0.00, 4.03)	0.87±1.05 (median: 0.55; 95%CI: 0.00, 3.99)
Playing with Electronic Gadgets (hours daily)	0.11±0.29 (median: 0.00; 95%CI: 0.00, 0.99)	0.38±0.50 (median: 0.25; 95%CI: 0.00, 1.71)	0.08±0.19 (median: 0.00; 95%CI 0.00, 0.71)	0.31±0.39 (median: 0.19; 95%CI 0.00, 1.37)	0.15±0.38 (median: 0.00; 95%CI: 0.00, 1.44)	0.49±0.61 (median: 0.29; 95%CI: 0.00, 2.54)

95%CI, 95% Confidence Interval;

In univariate analysis, elongation in axial length was significantly associated with older age, urban versus rural region of habitation, higher level of education of father and mother, mental versus physical occupation of father and mother, higher family income, myopia of father and mother, smoking of father, less outdoors time spent with leisure and less total time spent outdoors, more indoor time spent with studying, and more time watching television (Table 2). Elongation of axial length was not significantly associated with gender, higher body height and weight, birth weight, older mother’s age at birth, history of breast feeding, outdoors time spent with sports, self-reported smoking of the father and mother during pregnancy, alcohol consumption by the father or mother, and self-reported time watching television or playing with electronic gadgets (Table 2).

**Table 2:** Parameters Associated with the Elongation of Axial Length within One Year in Students of Grade 1 and 4 in Greater Beijing (Univariate Analysis).

Parameter	P-Value	Regression Coefficient BOr Mean Difference	Standardized CoefficientBeta	95% Conf. Interval of B or Mean Difference
Age (Years)	0.052	−0.03	−0.08	−0.05, 0.00
Gender	0.19	−0.05		−0.13, 0.03
Rural/Urban Region	<0.001	−0.19		−0.27, −0.11
Body Weight (kg)	0.85	0.000	−0.009	−0.005, 0.004
Body Height (cm)	0.99			−0.004, 0.004
Body Mass Index (kg/m^2^)	0.67	0.002	0.02	−0.006, 0.01
Parental Parameters				
Level of Education, Father	0.001	0.12	0.16	0.06, 0.18
Level of Education, Mother	0.001	0.10	0.14	0.04, 0.16
Occupation (Physical/Mental),				
of the Father	0.001	0.15		0.06, 0.24
of the Mother	0.006	0.14		0.04, 0.24
Father Myopic	0.007	0.14		0.04, 0.24
Mother Myopia	<0.001	0.18		0.08, 0.27
Behavior				
Outdoors Time with Sports	0.57	−0.10	−0.03	−0.45,0.25
Outdoors Time with Leisure	0.007	−0.09	−0.13	−0.15, −0.03
Outdoors Activity Total	0.01	−0.09	−0.13	−0.15, −0.02
Time Watching Television	0.03	−0.05	−0.10	−0.10, −0.004
Time Spent with Studying	0.005	0.07	0.13	0.02, 0.12
Time with Electronic Gadgets	0.81	0.02	0.01	−0.14,0.18

In a first step of a multivariate analysis, elongation in axial length was taken as dependent parameter and age and region of habitation as independent variables. It showed that axial length was associated with urban region of habitation (*P*<0.001). Adding the level of paternal and maternal level of education to the list of independent parameters showed that both parameters were not significantly correlated with the change in axial length. If, instead of the parental level of education, parental occupation was added to the list of independent parameters, maternal occupation and paternal occupation were both not significantly associated with elongation of axial length. Adding the parameters of parental myopia to the list of independent parameters revealed significant associations between elongation of axial length and maternal myopia (*P* = 0.02), while paternal myopia was not associated with elongation of axial length, after adjusting for region of habitation. After dropping the parameters for which the *P*-value was higher than 0.05 from the analysis, elongation of axial length was eventually associated with urban region of habitation (*P* = 0.001), and maternal myopia.

We then added all the activity parameters to the list of independent parameters and dropped step by step the parameters with the highest *P*-values from the list of independent parameters. After deleting region of habitation, time spent outdoors with sports, time spent outdoors with leisure, time spent indoors with watching television, and time spent indoors with playing with electronic gadgets sports, the increase in axial length was significantly associated with less total outdoors time (*P* = 0.02; standardized coefficient beta: −0.12), and more time spent indoors with studying (*P* = 0.007; beta: 0.14) in addition to maternal myopia (*P* = 0.02; beta: 0.12). Analysis of collinearity revealed for all parameters variance inflation factors of less than 1.05.

Out of the whole study population, 570 (88.6%) children showed an increase in axial length. In binary regression analysis, an increase in axial length was associated with time spent outdoors (*P* = 0.02; OR: 0.53; 95%CI: 0.32, 0.88), while urban region of habitation was not significantly associated (*P* = 0.52; OR: 0.72; 95%CI: 0.26, 2.04). If an increase in axial length of more than 0.25 mm (i.e. the median of the axial length increase) as compared to an axial length increase of ≤0.25 mm was taken as dependent variable, the analysis revealed a significant association with urban region of habitation (*P*<0.001; OR: 0.15; 95%CI: 0.09, 0.25), while less time spent outdoors was not significantly associated (*P* = 0.08; OR: 0.74; 95%CI: 0.53, 1.04).

### Increase in Axial Length/Anterior Corneal Curvature (AL/CC)

In univariate analysis, increase in AL/CC was significantly associated with urban versus rural region of habitation, higher level of education of father and mother, mental versus physical occupation of father and mother, higher family income, myopia of father and mother, less total time spent outdoors, less outdoors time spent with leisure, more indoor time spent with studying, more time watching television, older mother’s age at birth. Change of AL/CC was not significantly associated with age, gender, body height and weight, birth weight, history of breast feeding, self-reported smoking of the father and mother, alcohol consumption by the father and mother, time spent outdoors with sports and playing with electronic gadgets.

Multivariate analysis than included the change of AL/CC as dependent parameter and all non-activity variables as independent parameters which showed a significant association with the change in AL/CC in univariate analysis. It revealed that the change in AL/CC was significantly associated with paternal myopia and urban region of habitation. If then the activity parameters were added to the list of independent parameters, none of these parameters was significantly associated with the change in AL/CC. If the region of habitation was dropped from the analysis, the change in AL/CC was significantly associated with less time spent outdoors (*P* = 0.01; beta: −0.12) and paternal myopia (*P* = 0.003; beta: 0.15).

Out of the whole study population, 520 (80.9%) children showed an increase in the AL/CC ratio. In binary regression analysis, an increase in the AL/CC ratio was associated with urban region of habitation (*P* = 0.002; OR: 0.39; 95%CI: 0.21, 0.72) and mental versus physical occupation of the father (*P* = 0.006; OR: 0.56; 95%CI: 0.37, 0.85). If an increase in the AL/CC ratio of ≥0.03 (i.e. the median of the AL/CC increase) as compared to an AL/CC increase<0.03 mm was taken as dependent variable, the analysis revealed a significant association with urban region of habitation (*P*<0.001; OR: 0.20; 95%CI: 0.12, 0.32), while time spent outdoors was not significantly associated (*P* = 0.60).

### Change in Refractive Error

In univariate analysis, the increase in myopic refractive error was significantly associated with older age, urban versus rural region of habitation, body height and weight, higher level of education of father and mother, mental versus physical occupation of father, smoking by the father, higher family income, less total outdoors time, less outdoors time spent with leisure, more indoors time spent with studying. Progression of refraction was not significantly associated with gender, history of breast feeding, mother’s age at birth, myopia of father and mother, mental versus physical occupation of mother, self-reported smoking of the mother during pregnancy, self-reported alcohol consumption by father and mother, birth weight, outdoors time spent on sports, indoors time spent with watching television and time spent playing with electronic gadgets.

In a first step of the multivariate logistic analysis, change in refractive error was taken as dependent parameter, and independent parameters were age, region of habitation, body mass index, and body height. It showed that the increase in myopic refractive error was associated with older age and urban region of habitation. Adding the level of paternal and maternal education to the list of independent parameters showed that both parameters were not significantly correlated with the change in refractive error. If, instead of the parental level of education, paternal occupation was added to the list of independent parameters, paternal occupation was not significantly associated with progression of refraction. Adding family income and paternal smoking to the list independent parameters revealed both were not associated with progression of refraction, after adjusting for age, region of habitation.

We then added the activity parameters to the list of independent parameters and found that none of these parameters was significantly associated with progression of refraction (sport outdoors time, leisure outdoors time, total outdoors time, television indoors time, studying indoors time, electronic time) after adjusting for age, region of habitation. If region of habitation was dropped from the list of independent parameters, an increase in the myopic refractive error, after adjustment for age, was significantly associated with less time spent outdoors for leisure (*P* = 0.006; beta: −0.13), with less total time spent outdoors (*P* = 0.04; beta: −0.10), or with more time spent indoors with studying (*P* = 0.005; beta: 0.13).

## Discussion

In our longitudinal study on school children in Greater Beijing, a change in myopia related oculometric parameters such as axial length, the ratio of axial length to corneal curvature, and non-cycloplegic myopic refractive error, were significantly associated with less time spent outdoors and more time spent indoors after adjustment for systemic parameters such as parental myopia and age.

The mean increase in axial length 0.26±0.49 mm within one year corresponds with previous studies. Fan and colleagues investigated 307 preschool children with an age of 2–6 years and found a mean change in axial length of 1.72 mm within a 5 year period [26]. Lam et al reported that axial length increased by 0.51 mm in girls and 0.54 mm in boys aged 6–17 years during a two year period [27]. Saw and coworkers evaluated 543 myopic children aged 7–9 years and detected an annual axial length increase of 0.34 mm, 0.45 mm, and 0.10 mm in three years [28]. In the COMET (Correction Of Myopia Evaluation Trial) study, the increase in axial length in myopic children aged 6–11 years and using single vision lenses was 0.75 mm over 3 years [29].

The results of our longitudinal study agree with the findings from previous cross-sectional and longitudinal studies. In the longitudinal study by Jones and colleges, the effect of sports and outdoor activity hours per week on the development of myopia depended on the number of myopic parents [13]. Lower amounts of sports and outdoor activity increased the odds of becoming myopic in those children with two myopic parents more than in those children with either zero or one myopic parent. The chance of becoming myopic for children with no myopic parents appeared lowest in the children with the highest amount of sports and outdoor activity, compared with those with two myopic parents. In another study conducted by Jones and coworkers, eventually myopic children as compared with stable emmetropic children did not differ in near work activities at baseline, however had fewer outdoor/sports activity hours than the emmetropes [15]. After onset of myopia, outdoor/sports activity nor near work were associated with the amount of myopia progression [16]. In a similar manner, Onal and colleges reported that non-myopic children had a significantly higher prevalence of outdoor activity before and at age seven than did myopic children [14]. Parssinen and Lyyra reported that the factors with the most significant relationships to myopic progression were female gender, age of onset, and degree of myopia at the beginning of the follow-up, with myopic progression and final myopia being related to time spent on reading and close work [12]. The amount of time spent outdoors and with sports was connected with myopic progression and final refractive error in boys. In the study by French and colleagues, the time spent outdoors was negatively associated with incident myopia [20]. Near work and parental myopia were additional significant risk factors for myopia only in the younger cohort. Finally, the results of our investigation agree with the landmark study by Rose and colleagues who reported that higher levels of total time spent outdoors were associated with less myopia and a more hyperopic mean refraction, after adjusting for near work, parental myopia, and ethnicity [7].

In our study, the mean elongation of axial length was 0.26±0.49 mm (95%CI: −0.70, 0.89) and the mean increase in AL/CC was 0.03±0.06 (95%CI: −0.08, 0.12). These data were comparable with the results obtained in the study by Donovan and colleagues who found a mean axial elongation of 0.17±0.10 mm for summer, 0.24±0.09 mm for autumn, 0.24±0.09 mm for winter, and 0.15±0.08 mm for spring [17]. In a similar manner, Fujiwara and co-workers found a mean axial elongation of 0.14±0.01 mm for summer, 0.17±0.01 mm for winter and 0.16±0.01 mm for the other seasons [18]. A recent study was performed by Cui and colleagues in Denmark where due to the northern location the length of the day varies over the year from 7 to 17.5 hours. The authors found significant correlations between the hours of daylight and eye elongation, myopia progression, and corneal power change, so that they concluded that children may be encouraged to spend more time outside during daytime to prevent myopia [23]. The results from Asia thus match data from Europe. In agreement with the previous studies, Sherwin and colleagues found that an increasing area of conjunctival ultraviolet autofluorescence, a biomarker of subacute outdoor light exposure, was protectively associated with prevalent myopia [9]. Interestingly, French and coworkers observed that European Caucasian children in Northern Ireland, with a marked different light exposure as compared to children in countries closer to the equator, had a greater prevalence of myopia, hyperopia, and astigmatism when compared to children living in Sydney [11]. Known risk factors for myopia such as parental myopia, parental education and educational standards did not explain the differences. Further work on levels of near work and time spent outdoors is required.

It has remained unclear why a higher amount of outdoor activities in contrast to a higher amount of indoor studying was associated with less axial length elongation and lower increase of the AL/CC ratio. In previous studies and reports, different mechanisms and reasons for the association between myopia or development of myopia and outdoors activity have been discussed in detail. These mechanisms and reasons included the potential influence of dim illumination during reading or light intensity in general on the development of myopia, the potential dopamine-dependent effect of bright light (socalled “light-dopamine hypothesis”) preventing the development of myopia in animal models, the influence of exposure to bright light, viewing distances, and others [10], [30]–[37]. In a recent survey, French and coworkers reviewed the potential mechanisms associated with outdoors activities and prevention of myopia [32]. They summarized that a light-stimulated release of dopamine from the retina may be involved, since increased dopamine release appeared to inhibit increased axial elongation. That hypothesis was supported by experimental investigations in which the D2-dopamine antagonist spiperone appeared to partially reduce the protective effect of bright light [37].

Future research may primarily address the physiologic mechanism governing the precise growth and elongation of the eye in very fine relationship to the main parameters of the optics of the eye, such as corneal curvature, lens position, anterior and posterior lens curvature, and axial length. If one has discovered the sensor detecting whether the eye is a bit too short or too long, the effector, which influences or governs the elongation of the globe, and the communicating system between sensor and effector, one may then be able to stop an abnormal elongation of the globe, medically or physically. Until then, one may address the research on extraocular factors such as the behavior of the children and of the parents how these parameters influence the elongation of the eyes and how one can influence theses mechanisms, by measures such as spending more time outdoors. Other research may be directed on the question which aspects of spending outdoors may be of importance, such as the amount of ultraviolet light, the brightness in general, the length of the day and night, the ability to see the far horizon, or the activities performed when being outdoors. Previous studies were focused on the effect of single vision versus bifocal or progressive addition lenses, of contact lenses or the use of atropine on myopia progression [38]–[45].

The results of our study in association with the findings obtained in the previous investigations may have public health ramifications. The school agenda in particular in the large cities may be changed in the sense that the school children spend considerably more time outdoors during school time than they have been spending so far. Future studies may address whether very large windows in school rooms could also be helpful in preventing the development of progression of myopia in school children. And one may examine whether using the top roof of school buildings for school rooms as long as the climate and the architecture allow it may be another useful measure to stop the myopic shift in the young population living at the Pacific rim and in large Inner-Chinese cities.

Potential limitations of our study should be mentioned. First, the study was not population-based so that the possibility of a selection bias existed. Since it was a follow-up study, the potential disadvantage of a non-population-based study design may have been less prevalent than for a cross-sectional study design. Second, refractometry was not performed under cycloplegic conditions, so that involuntary accommodation during refractometry will have covered latent hyperopia. The main outcome parameters of our study were, however, axial length and the AL/CC ratio the measurement of which is independent of the accommodative status of the lens. Without doubt, the biometric measurements as compared to the refractometric data have a much higher value for this study, and the use of non-cycloplegic refractometric study in this study may not encourage to use such data without biometric measurements. Third, the data on the time spent for various activities were self-reported. Fourth, a follow-up of one year was relatively short. Interestingly, however, the association between axial length elongation and outdoor activity was statistically significant despite the short follow-up so that this weakness in the study design may serve to strengthen the conclusions of the study. Fifth, the list of independent variables in the multivariate analysis included various parameters which were significantly associated with each other. These parameters include the activity measures and the region of habitation. Correspondingly, the increase in axial length or in the AL/CC ratio was associated with either urban region of habitation or less time spent outdoors. To avoid a potential bias due to a confounding effect, we additionally performed an analysis of collinearity in which the variance inflation factors were less 1.05.

In conclusion, a change in oculometric parameters indicating an increase in myopia was significantly associated with less time spent outdoors and more time spent indoors in school children in Greater Beijing within a study period of one year. Our longitudinal study provides additional information on the potentially helpful role of outdoors activity in the prevention of myopia. Public health care measures are necessary such as that school timetables take account of the potential for increased time outdoors to prevent the onset of myopia. Future studies may potentially also address whether very large windows in school rooms could also be helpful in preventing the development of progression of myopia in school children.
